# A task-unified network with transformer and spatial–temporal convolution for left ventricular quantification

**DOI:** 10.1038/s41598-023-40841-y

**Published:** 2023-08-19

**Authors:** Dapeng Li, Yanjun Peng, Jindong Sun, Yanfei Guo

**Affiliations:** 1 Shandong University of Science and Technology, Qingdao, China; 2 Shandong Province Key Laboratory of Wisdom Mining Information Technology, Qingdao, China

**Keywords:** Bioinformatics, Biological models

## Abstract

Quantification of the cardiac function is vital for diagnosing and curing the cardiovascular diseases. Left ventricular function measurement is the most commonly used measure to evaluate the function of cardiac in clinical practice, how to improve the accuracy of left ventricular quantitative assessment results has always been the subject of research by medical researchers. Although considerable efforts have been put forward to measure the left ventricle (LV) automatically using deep learning methods, the accurate quantification is yet a challenge work as a result of the changeable anatomy structure of heart in the systolic diastolic cycle. Besides, most methods used direct regression method which lacks of visual based analysis. In this work, a deep learning segmentation and regression task-unified network with transformer and spatial–temporal convolution is proposed to segment and quantify the LV simultaneously. The segmentation module leverages a U-Net like 3D Transformer model to predict the contour of three anatomy structures, while the regression module learns spatial–temporal representations from the original images and the reconstruct feature map from segmentation path to estimate the finally desired quantification metrics. Furthermore, we employ a joint task loss function to train the two module networks. Our framework is evaluated on the MICCAI 2017 Left Ventricle Full Quantification Challenge dataset. The results of experiments demonstrate the effectiveness of our framework, which achieves competitive cardiac quantification metric results and at the same time produces visualized segmentation results that are conducive to later analysis.

## Introduction

Cardiovascular diseases (CVDs) are the leading cause of death globally according to World Health Organization (WHO), about 17.9 million people died from CVDs in 2016, from CVDs, mainly from heart disease and stroke^1^. CVDs is a general term for a series of diseases caused by heart and blood vessels, such as coronary heart disease, stroke, heart failure, rheumatic heart disease, congenital heart defect, and arteriovascular disease. In recent years, with the rapid development of society and economy, people’s lifestyles have undergone profound changes. Due to unheathy living habits, aged tendency population, and the continuous prevalence of the metabolic syndrome, the incidence of cardiovascular diseases is in a continuous upward stage. Cardiovascular diseases are currently showing a sudden and youthful trend, requiring timely detection and treatment of the disease. The heart is the most important organ of the human body, whose main function is to provide power for blood flow, transport blood to various parts of the body, and maintain normal metabolism and function of cells. The abnormality of the shape, volume and functional parameters of the heart is a sign of various CVDs. For example, an abnormal shape of the heart is a symptom of hypertrophic heart disease, abnormal volume is a characteristic of dilated cardiomyopathy, enlargement of left atrium and Right ventricle is a sign of rheumatic heart disease, the gradual decrease of left ventricular ejection fraction is an important feature of coronary heart disease. Therefore, monitoring the shape, volume and function of the heart through medical instruments has become the most important way to diagnose and treat cardiovascular diseases. In specific clinical applications, imaging equipment is used to obtain a patient’s heart image. Imaging doctors annotate the anatomical structure of the heart, quantify the cardiac metrics, and provide assistance for the next step of diagnosis and treatment.

In order to provide support for the diagnosis and curing of the CVDs, considerable medical imaging technologies, including computed tomography (CT) and magnetic resonance imaging (MRI) are exploited. Cardiac MRI has a good contrast resolution of soft tissues, a large scanning field of view, and can obtain oblique cross-sectional images in various directions and different angles. It has become the gold-standard for non-invasive and non-radiative evaluation of cardiac structures and functions^2^. Left ventricle (LV) quantification indices such as end-diastolic internal meridian, end-systolic internal meridian and ejection fraction (EF) are the most important indicators for evaluating the cardiac function in clinical practise. Therefore, the accurate quantification of clinical cardiac functions is of great importance for helping early diagnosis and identification of CVDs.

In the clinical approach, LV function information relies on the manually laborious delineation of the LV epicardium and endocardium laborious by radiologists. Meanwhile, human assessment of LV function has changeable anatomy structure in systolic diastolic cycle and the laborious nature of a calculation that hard to trace^3^. So with regard to LV quantification, although many efforts have been devoted to find automatic or semi-automatic methods to solve above problems, the following challenge issues should be addressed for robust and accurate LV quantification: (1) the variability of cardiac ventricle in shape and appearance in whole cardiac cycle frame sequences due to different pathologies. (2) the low contrast anatomy structures, in-homogeneity brightness and texture in MRI^4,5^.

**Figure 1:**
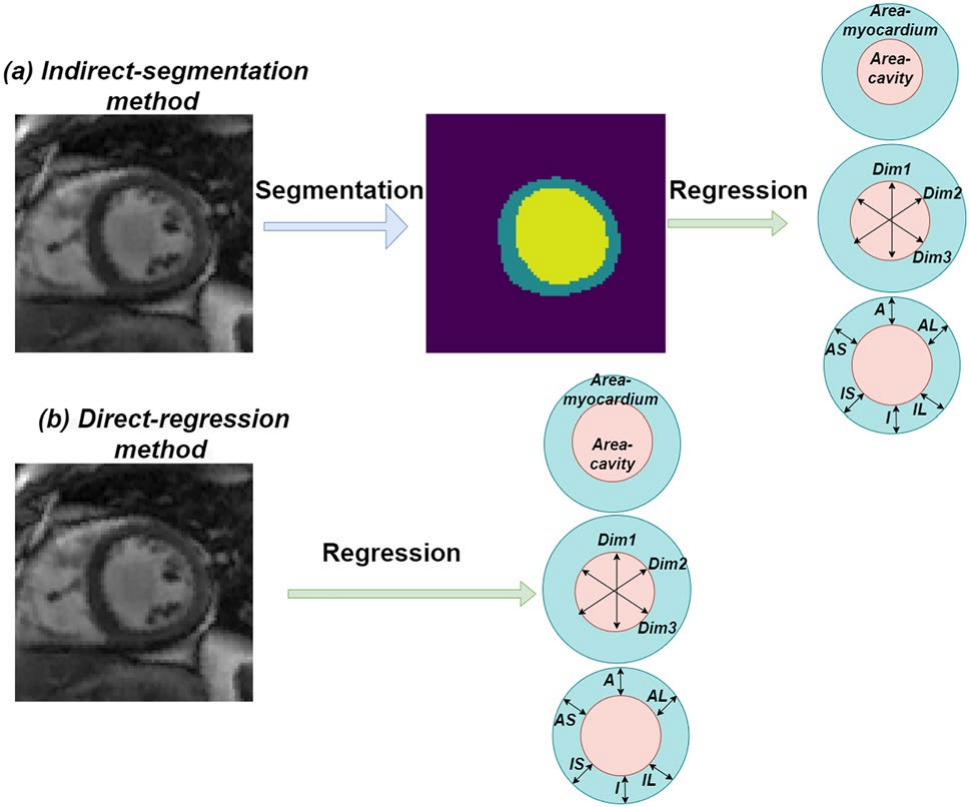
Two categories methods have existed in left ventricular quantification domain. (**a**) Segmentation-based methods compute indices from the segmented result which requires strong prior information and user interaction. (**b**) Existing direct regression methods of cardiac indices quantification. When the labeled image is not available, direct methods-regression without segmentation step have grown in popularity in cardiac LV indices estimation.

Doctors are used to draw the structural contour of cardiac LV cavity and LV myocardium manually in early clinical practice, they use the segmented contour to obtain the reliable quantification. However, due to the large number of cardiac images, this process is still time-consuming and tedious. Therefore, exploring automated methods to reduce the laborious work of radiologists and increase the precision of quantification is of great importance. Two categories methods have existed in left ventricular quantification domain, those are the indirect-segmentation based method and the direct-regression method (as depicted in Fig. 1). Although these models have showed great performance in cardiac LV quantification, both of the above two methods have advantages and disadvantages. By integrating segmentation module and regression module into a uniform platform will help the framework to exploit more robust feature representations and achieve precise quantification results. Considerable of methods have been introduced in cardiac quantification field, Xue et al.^2^ proposed a Bayesian neural network incorporate the Monte-Carlo dropout for deep feature extraction, then they designed an uncertainty weighted loss function train the network. Du et al.^6^ utilized a two step network which consists a segmentation network to achieve the contour of target and a regression network to quantify LV indices based on the previous segmentation results. Vesal et al.^7^ first segmented cardiac LV contour using an encoder-decoder architecture network, and then introduced a multi-task framework that consists of regression task and classification task to achieve the final results. Ge et al.^8^ raised a K-shaped Unified Network to direct segment and quantify LV simultaneously. Chen et al.^9^ utilized dynamic analysis module, segmentation module, and quantification encoder module to make up a multi-task conditional learning model.

Although these elaborately designed approaches improve the generalization performance, some aspects of disadvantage should not be neglected. As to the muti-module network, the feature information from the segmentation path is not enough exploited, complex multi-module network is susceptible to degrade quantification performance as a result of the degrade segmentation performance. In this paper, a new end-to-end fully automatic deep learning segmentation and regression task-unified framework for LV segmentation and quantification is proposed. The task-unified model, which consists of a segmentation path and a regression path, help to represent origin image, learn multi-scale features and seize cardiac anatomy structural spatial–temporal information. Through this method, LV function can be acquired through the final regression learning network and provide clinicians with quantitative diagnosis.

As such, the contributions of this work are summarized as follows: (1) A robust and effective task-unified framework to improve the performance of complete LV indices quantification, which includes two areas, three cavity sizes, six regional wall thicknesses, (2) Leverage the segmentation network to obtain visual segmentation results and provide reconstruct low noisy feature maps for regression network. (3) A combination multi-task loss is used to supervise the unified framework.

We conduct fivefold cross-validation experiment on the public MICCAI-2018 Left Ventricle Full Quantification Challenge (LV-Quan) dataset . Results of the cross-validation experiments demonstrate the competitive performance. The remainder of this paper is organized as follows. In “Related works” section, related works in cardiac ventricle quantification field is given. “Methods and materials” section presents our proposed multi-task deep learning segmentation and regression unified framework architecture. The segmentation and quantification experimental results are detailed in “Experiments and results” section. Finally, the conclusion is presented in “Conclusions” section, and acknowledgement is presented in “Acknowledgements” section.

## Related works

### LV quantification methods

Indirect-segmentation methods segment the LV myocardium first and then quantify the cardiac indices. Direct-regression methods exploit the mapping relations between the cardiac MR images and cardiac indices directly. Owing to the powerful representation ability of neural networks, both of those methods have improved the performance for quantification of cardiac LV indices.

The indirect-segmentation based method is a two-step approach which the desired cardiac LV indices of the second step are measured based on the segmentation results of the first step. Most of the early LV quantification works^10,11^ fall into this category. Classic image processing methods such as active contour^12,13^, level-set^14^, deformable model and prior knowledge have gained great development in the past decades^15,16^. Recently, convolution neural networks (CNNs) have showed impressive performance for segmenting cardiac LV by level set and deformable model^17–20^. Other deep neural network architectures introduced in cardiac segmentation field including parallel coarse-to-fine network^21^, grid-like CNN^22^, encoder-decoder architecture^23^, dilated CNN^24^, deep supervision 3D-CNN^25^, generative adversarial learning^26^, and shape prior knowledge^27^. Zhen et al.^28^ used multi-scale deep neural network to learn hierarchical information initially and then put them into random forest to regression the cardiac LV indices.Furthermore, they proposed supervised descriptor learning to calculate four chamber volumes^29^. Wang et al.^30^ leveraged an adaptive Bayesian method combining with shape features to estimate ventricular cavity volumes. The indirect-segmentation methods can offer not only the cardiac indices quantification results, but also the visualization results of the cardiac LV myocardium. However, in this category methods, it is a cascade approach which have only forward connection but no feedback from the second step. As a result, the unrepresentative extracted features will results unaccurate quantification results.

The direct-regression method for cardiac LV quantification has go through considerable development and recognition^31–35^. When the annotated groundtruth of image is not provided, direct methods-regression is a preferable method. This method can enable many effective analyze tools on cardiac MRI^28^. As direct architecture facilitates to seize more expressive LV information, the combination of feature representation and regression models are introduced. Luo et al.^36^ estimated the cardiac volume by leveraging a multi-views fusion strategy in cardiac systole and end diastole cycle. Kabani et al.^37^ used CNN to crop ROI, estimate volume from cardiac systole and end diastole cycle. Xue et al.^38^ introduced the first end-to-end cardiac indices quantification framework. Additionally in^39^, they used a multitask neural network, which mapped the relations among cardiac LV indices and between tasks by Bayesian-based relationship learning. Although these methods demonstrated their effectiveness, there are still difficulties for the direct-regression methods to learn representative features due to highly variable cardiac anatomy structures.

### Cardiac quantification indices

**Figure 2:**
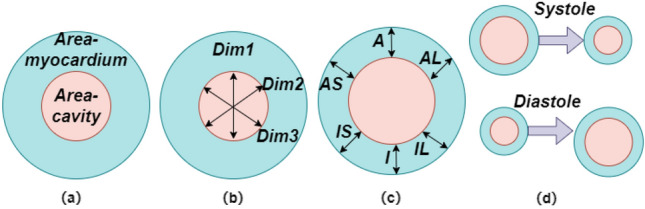
Schematic illustration of LV indices for short-axis cardiac MRI. (**a**) The LV and Myocardium cavity areas are shown with blue and pink color. (**b**) LV cavity directional dimensions with black arrows. (**c**) Six myocardial regional-wall thicknesses (RWT), namely anterolateral (AL), inferolateral (IL), inferior (I), inferoseptal (IS), anterior (A), inferoseptal (IS) and anteroseptal (AS). (**d**) The cardiac phase (systole or diastole).

The quantitative indices of the cardiac LV mainly include the six regional wall thickness of LV myocardium and LV cavity that describe anatomical structural information, and LV cavity and myocardium areas that used to calculate cardiac function parameters such as ejection fraction (EF). As demonstrated in Fig. 2. The cardiac metrics are strongly correlated with regional and cardiac function assessments. In^10^, the clinical roles of more cardiac indices are fully explained. Many existing methods focus on estimate the LV volume, which is simplified to the integral of the cavity area or is hard to quantify as a result of the high contrast. When multi-type cardiac quantification indices are estimated, more challenges would be arise. On the one hand, the cardiac quantification indices are different from each other in relation to the 2D spatial image structure, so a more robust and relevant representation is needed for estimation. On the other hand, in terms of LV indices, regional wall thickness and myocardial area are suffer from the complex dynamic deformation of the myocardium, as well as the invisible cardiac ventricular epicardial edge. The regional wall thickness is also affected by the orientation of the myocardial. Thus the segmentation and regression paths should be able to sustain dynamic deformation, imperceptible boundary and direction changes^38^. The LV-Quan dataset was held in conjunction with the Statistical Atlases and Computational Modeling of the Heart (STACOM) workshop at MICCAI^11^, which created a foundation dataset for researches on cardiac LV quantification.

## Methods and materials

**Figure 3:**
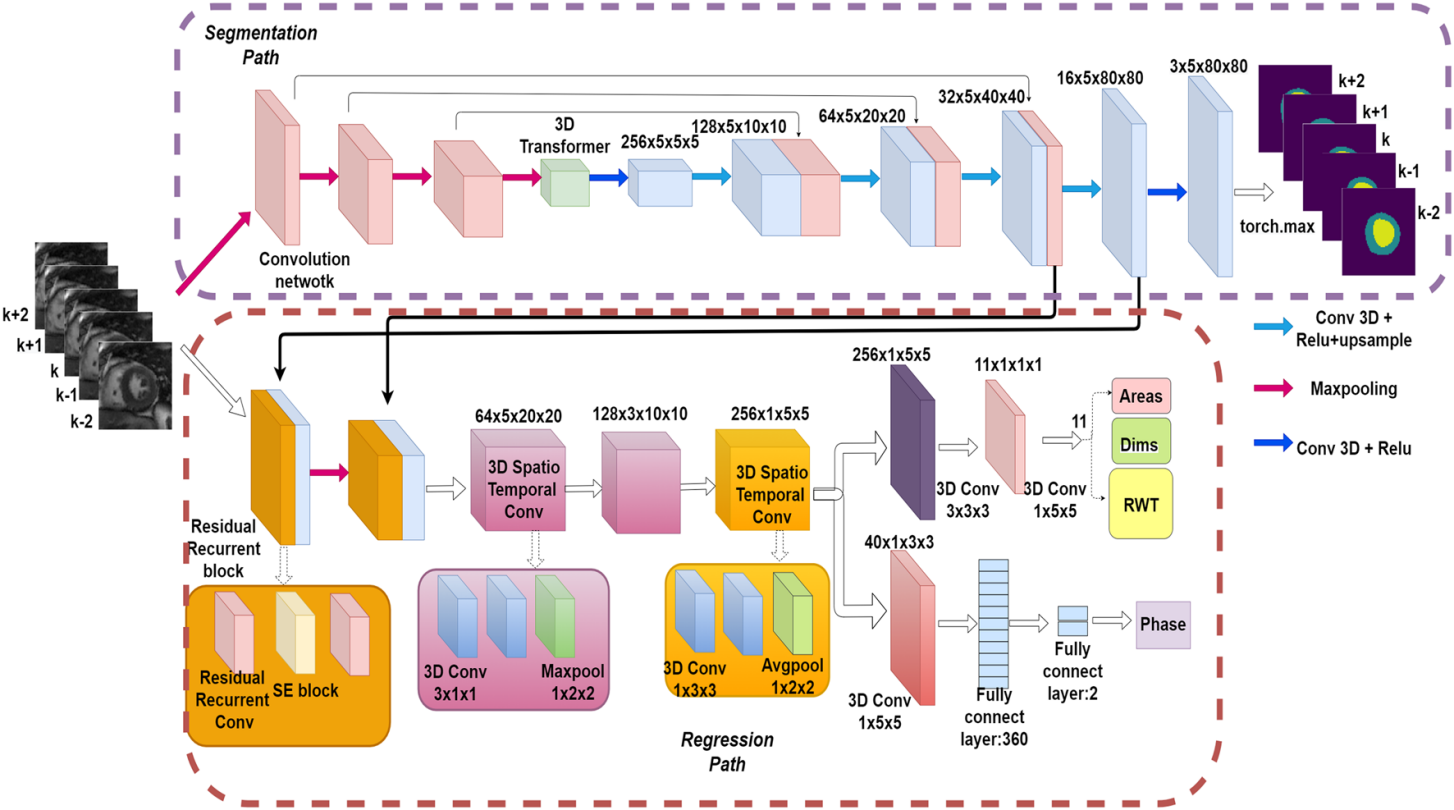
Overview of the proposed unified framework which contains segmentation path and regression path.

Overview architecture of the our task-unified framework is presented in Fig. 3. The cardiac LV indices quantification adopts the idea from direct regression methods. However, the mapping relation between the input cardiac MRI and the ground-truth label indices is fuzzy, we introduce a task-unified framework that propagates structural feature information from the previous segmentation path to the regression path in multi-scale. This framework takes sequences of 5 slices as a 3 dimension input, the segmentation path outputs prediction of 5 slices while the regression path predict the groundtruth indices for the middle slice. The framework is beneficial in three aspects: (1) We incorporate temporal dynamics feature information from the neighbor slices, thus alleviating the segmentation predict task. (2) Multi-scale structural image information from segmentation path enhance the ability of regression path for better cardiac LV quantification. In the following, details of the main components of the our framework are describe. (3) The unified framework reduces over-fitting and provides not only segmentation results but also quantification results.

### Segmentation path

To segment the LV cavity and LV myocardium from cardiac MRI, we employ a 3D Transformer U-Net architecture inspired by TransUNet^40^, which which merits both Transformer and U-Net. CNN-based framework have limitations of modling long range interactive feature, while Transformer have powerful long range modeling ability. So we use transfomer model in our framework. The Vision Transformer conduct as an encoders in segmentation path, and combine with U-Net to extract more finer spatial information. Vision Transformer^41^ is the foundation work and showed better performance especially for target structures that show large inter-patient variation in terms of texture, shape and size. The variability of cardiac LV in shape and appearance in whole cardiac cycle frame sequences due to different pathologies, so Transformer is strong recommended for cardiac image segmentation. And taking into account the temporal dynamic in cardiac systolic and diastolic cycle, we leverage the TransUNet network the replace the 2D convolution to 3D convolution. Our proposed 3D TransUNet is a u-shaped architecture, which hybrid CNN-Transformer is used as encoder to learn global context information as well as a cascaded CNN upsampler is employed to extract detail different scale spatial information for precise localization.

The input tensor size is Batchsize × Channel × Number of slices × Height × Width (20 × 1 × 5 × 80 × 80 ), we first use a resnet as feature extractor to generate a feature map for the input. The resnet has three downsample stages, and each stage has 3D convolution layers with kernel size of 3. Group-normalization, Random leaky Rectified Linear Unit, and a 3D max-pooling with kernel size of 1 × 2 × 2 are also used in each stage to decrease the feature dimensions. Finally the resnet output an image tensor with size of 2 × 256 × 5 × 5 × 5. Then transformer performed to encode the patch spatial information and output a tensor with size of 2 × 125 × 256. A cascaded upsampler is introduced as decoder, which consists of trans-convolution stages to decode the hidden layer feature information. we instantiate the cascade upsampler by reshaping the sequence of hidden feature and cascading trans-convolution blocks for ascent to full resolution that coincidence with the original image.

### Regression path

To regress the LV indices, we introduce a task-unified spatial–temporal convolution architecture, which is trained in indirect and direct approach simultaneously. This regression path consists of 3D spatio-temporal convolution blocks, Recurrent Residual Attention Convolutional (RRAconv) blocks and fully connection (FC) layer. Many previous works have used 3D spatio-temporal convolution block to incorporate spatial information and temporal dynamic information^42–44^. We employ 3D RRAconv to 2D + time image frames to learn temporal dynamic information. Each RRAconv block contains two Recurrent Residual convolution and a SE channel attention module. According to our understanding, noise in the original Cardiac MRI affect accuracy of regression. Hence, we add skip connection between multi-scale structural image information in segmentation path and LV indices information in regression path to release the original noise and improve the accuracy of quantification. SE is used to adaptively concatenate information from the current regression path and corresponding information from the segmentation path.

The input tensor of the regression path is a size of k × h × w, where k is the number of slices that indicate temporal dimension, and h × w denotes the spatial dimension. Each 3D RRAconv block has Recurrent Residual convolution with kernels size of 3. ReLU activation and 3D batch normalization are used in this block. The spatial–temporal block is composed of two cascade 3D convolution layers, and follow by a 3D Max-Pooling layer. In the two two cascade 3D convolution layers, previous layer use 3 × 1 × 1 kernel convolution to capture temporal information and the latter layer leverage 1 × 3 × 3 kernel convolution with strides of 1 to learn spatial information. The following Max-Pooling layer use 1 × 2 × 2 kernel to decrease the feature maps along the spatial dimension and temporal dimension to regression LV indices only for central slice. ReLU activation and 3D batch normalization are also used in this block. We initialize the convolution layer kernels with the He initializer and apply weight regularization to reduce the over-fitting problem^45^.

Since the previous segmentation path, RRAconv block and spatio-temporal block have extracted excessive representation information form cardiac MRI, there is no need to design a more complex or deeper neural network for the final multi-task of regression and classification. Finally, two parallel branches are derived to complete the final multi-task. One shallow CNN branch used as a regressor to quantify wall thickness, dimensions, and areas, another branch is a fully connected layer which composed of 360 neurons multi-layer perceptron, and an output neurons with 2 neurons to classify the cardiac systole or diastole phase.

### Loss function

Based on the two path of task, in this work, multi-task needs to be addressed and loss function should be elaborately designed to supervise the unified network. Therefore, we leverage joint-task loss function for both LV segmentation, indices regression and phase classification.

For the segmentation path, to segment a cardiac MRI with having LV myocardium, LV cavity and background as labels. An objective function optimizer was introduced for precise segmentation and prompt the network to tackle highly class imbalance problem. We employ a loss function that combine the Dice loss and Cross-Entropy (CE) loss. The Dice loss function can improve the segmentation metrics, and the CE loss can increase the accuracy. Many works have combined these two loss functions to supervise the neural network, and achieved impressive performance^46^. Motivated by this, we also combine these two loss function to construct a new loss. Since Dice loss puts more emphasis on the overall similarity coefficient,we empirically set weight $\lambda _1 = 1$ and $\lambda _2 = 1.5$ to each of the two loss functions. The overall loss function can be seen in Eq. (1).

In the regression path, we minimize a combination of Mean Squared Error (MSE) and binary cross-entropy (BCE) loss over sets of k slices where groundtruth annotations are only offer for the middle slice. Given a set of k slices $x_{i}$ = ($x_{0}$, ...,$x_{k-1}$ ), the label for the middle slice $y_{i}$ = ($y_{dim}$, $y_{area}$,$y_{rwt}$,$y_{phase}$ ) predictions of our model ${\hat{y}}$= ${\hat{y}}_{dim}$, ${\hat{y}}_{area}$,${\hat{y}}_{rwt}$,${\hat{y}}_{phase}$ the combination loss function is defined as Eq. (2). Equation (3) can be used to train the entire unified framework which consists of the segmentation path loss $L_{Seg_path}$ and regression path loss $L_{Reg_path}$ in an end-to-end approach. We have empirically set $\lambda _3 = 4$ and $\lambda _4 = 1$ as weights in Eq. (3) to weight importance and gradients of different task path. Since the regression path rely on the segmentation results, we give more weights to segmentation path task than to regression path task. This approach prompt the unified framework to output precise LV prediction.

1 $$\begin{aligned} L_{Dice}({\hat{y}},y) & = 1-\frac{2}{K}\sum _{K=0}^{K-1}\frac{\sum _{i}^{\Omega }y^{k}{\hat{y}}_{i}^{k}}{\sum _{i}^{\Omega }y^{k}+{\hat{y}}_{i}^{k}} \end{aligned}$$

2 $$\begin{aligned} L_{CE} & = -\frac{1}{|N|}\sum _{i}\sum _{c=1}^{K}y_{ic}log(P_{ic}) \end{aligned}$$

3 $$\begin{aligned} L_{Seg} & = \lambda _1L_{CE}+\lambda _2L_{Dice} \end{aligned}$$

4 $$\begin{aligned} L_{BCE}({\hat{y}},y) & = \frac{1}{|\Omega |}\sum _{i}^{\Omega }-y_{i}log({\hat{y}}_{i})-(1-y_{i})log(1-{\hat{y}}_{i}) \end{aligned}$$

5 $$\begin{aligned} L_{MSE} & = -\frac{1}{|N|}\sum _{s=1}^{11}\sum _{i=1}^{N}|| y_{s,i}-{\hat{y}}_{s,i} ||^{2} \end{aligned}$$

6 $$\begin{aligned} L_{Reg} & = L_{MSE}(y_{indices},{\hat{y}}_{indices})+ L_{BCE}(y_{phase},{\hat{y}}_{phase}) \end{aligned}$$

7 $$\begin{aligned} L_{Unified} & = \lambda _3L_{Seg}+\lambda _4L_{Reg} \end{aligned}$$

## Experiments and results

We implement our framework with PyTorch and he experiments were carried out on one NVIDIA RTX 2080TI GPU. The experiment results are presented in the following sections.

### Data and preprocess

The data used in this study includes 2900 cardiac MRI of 145 patients^38^. Every subject, have mid-cavity 20 frames in one cardiac systolic diastolic cycle. These images are from three affiliated hospitals of two medical centers (London Medical Center and St. Joseph’s Medical Center). The age of the subjects ranged from 16 to 97, with an average age of 58.9 years. The pixel spacing of MR images range from 0.6836 mm/pixel to 2.0833 mm/pixel, with the mode of 1.5625 mm/pixel. The pathological types of the subjects are diverse, including regional wall motion abnormalities, myocardial hypertrophy, mildly enlarged LV, atrial septal defect, LV dysfunction, etc. In each frame of image, the LV has three equal parts, that is the basal, mid-cavity, and apical^47^. Before the experiments, several pre-processing approaches are employed by the challenge organizer, which including (1) Landmark labelling. (2) Rotation. (3) ROI cropping. (4) Resizing. After this procedure, the images from different subjects are approximately aligned in size, orientation, and scale. Thus making the assessment independent of various pre-processing and allowing researchers to focus on the LV quantification.

In the ground-truth, LV myocardium epicardium and LV myocardium endocardium borders were manually labeled by radiologists. According to this border, we re-divide ground-truth into three category labels, those are being the LV cavity, LV myocardium and background. LV indices and cardiac phase is a great correlation with cardiac function metrics such as ejection fraction. The LV indices values are normalized by the dimension of the image or the pixel number.

**Figure 4:**
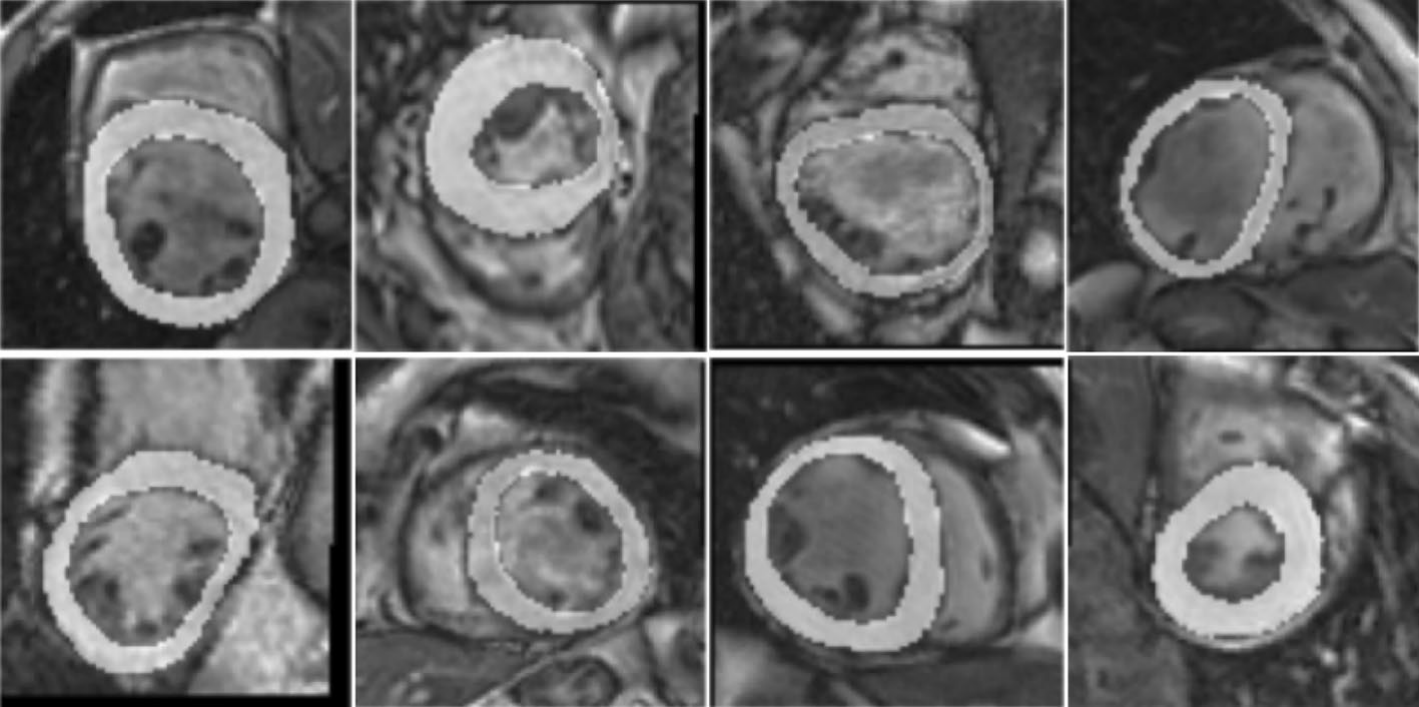
After pre-processing, we the stack the input image and its corresponding groundtruth to highlight the LV cavity and LV myocardium. From the figure, we can see the variation of shape, contrast and density in cardiac MRI. It is a great challenge for segmentation and quantification.

We conduct five-fold cross-validation experiment on the LV-Quan dataset. We first use z-score normalization which based on the mean and standard deviation value and then employed data augmentations techniques including elastic random rotations transformation between − 90 and 90°, random horizontal and vertical flips transformation with chance of 50 percen, elastic deformations transformation, and gamma shifts transformation with the scope of 0.5 to 1.5. Contrast Limited Adaptive Histogram Equalization (CLAHE) is applied to each training image slices to weaken the intensity inhomogeneity problem (as is shown in Fig. 4). In the training strategy, the segmentation model was trained with RAdam optimizer for 500 epochs with $\beta _1$ = 0.9 and $\beta _2$ = 0.999, along with weight decay value of 1E−4, and initial learning rate of 5E−4 exponentially decayed with parameter 0.99. The transformer module are pre-trained with ImageNet^48^. The regression model and classification model both using SGD optimizer with a learning rate of 5E−4, and with weight decay rate of 5E−3 and momentum parameter 0.06.

8 $$\begin{aligned} Dice(A,B) & = \frac{2|A\bigcap B|}{|A\bigcap B|+|A\bigcup B|} \end{aligned}$$

9 $$\begin{aligned} Hausdorff(A,B) & = max[h(A,B), h(B,A)] \end{aligned}$$

### Results

10 $$\begin{aligned} MAE_{indices} & = \frac{1}{|N|}\sum _{i=1}^{N}| {\hat{y}}_{indices}-y_{indices}| \end{aligned}$$

11 $$\begin{aligned} PCC_{indices} & = \frac{\sum _{i=1}^{N}({\hat{y}}_{indices}-{\overline{y}}_{indices})({y}_{indices}-{\overline{y}}_{indices})}{\sqrt{\sum _{i=1}^{N}({\hat{y}}_{indices}-{\overline{y}}_{indices})^{2}({y}_{indices}-{\overline{y}}_{indices})^{2}}} \end{aligned}$$

12 $$\begin{aligned} MAE_{indices} & = \frac{1}{|N|}\sum _{i=1}^{N}| {\hat{y}}_{phase}\ne y_{phase}| \end{aligned}$$

The performance of out task-unified model is evaluated in terms of prediction accuracy of LV segmentation and LV indices quantification. Dice and Hausdorff Distance metrics are used to evaluate the performance of the LV segmentation. The Dice Coefficient metric is defined Eq. (4). Evidently, Dice(A,B) is maximized at 1 when A = B and minimized at 0 when A $\ne$ B. where A and B are two sets. The Hausdorff Distance metric is defined in Eq. (5): where h(A,B) represents the distance from point A to point B. In addition, we leverage the mean absolute error (MAE) , Pearson correlation coefficient (PCC) and Error Rate to evaluate the regression path performance . They defined as Eqs. (6), (7) and (8), where ${y}_{indice}$ is the the ground-truth label indices and ${\hat{y}}_{indice}$ is the predicted value of indices by our proposed unified framework. Here, ${\overline{y}}_{indice}$ and $\overline{{\hat{y}}}_{indice}$ is the mean value. ${\hat{y}}_{phase}$ and $y_{phase}$ are the label annotation and predict class for the cardiac systolic diastolic phase.

We report the performance of our model below including performance of LV segmentation path and performance of LV quantification path.

#### Performance of LV segmentation path

**Figure 5:**
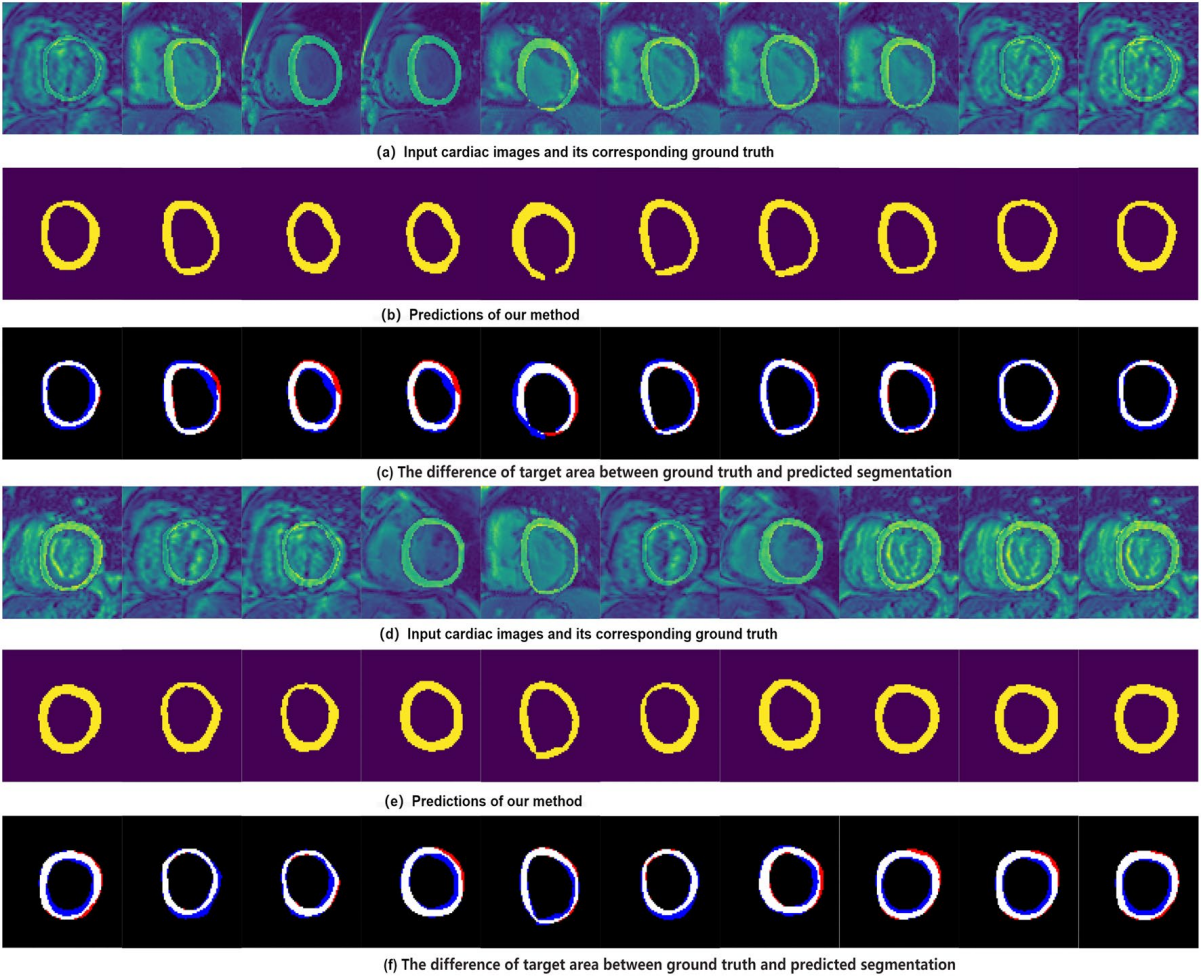
Example of predictions by our model for a random 20 frames: The rows of (**a**) and (**d**) are input images and corresponding myocardial, (**b**) and (**e**) are the myocardial predictions from our unified network. The rows of (**c**) and (**f**) are the difference of target myocardial area between ground truth and predicted segmentation, where red represents the under segmented regions and blue indicates the over segmented regions.

Segmentation is one of our tasks and segmentation path is also used as a structural feature extractor for regression path.To verify that segmentation path can aggregate representative structural information and output predictions that most closely resemble the correct results. We use Dice and HD metrics to evaluate performance of our proposed segmentation model by comparing it with classic segmentation methods including UNet^49^, Densenet^50^, IndicesNet^38^, MC-Seg^51^, DRUNet^7^, Parallel^52^ and SAUNet^53^. Dice Coefficient metric and HD metric are reported in the Tables 1 and 2. we can conclude that each method show competitive performance, our proposed method outperform other method in LV myocardium segmentation performance and the segmentation performance of LV cavity is better than that of LV myocardium. LV cavity and LV myocardium is the region of interest, which suffer shape variation during a cardiac systolic diastolic cycle and across different data subjects. It is difficult to recognize these two class labels, especially LV myocardium. The qualitative segmentation predictions of our framework are showed in Fig. 5. The first row are input images and its corresponding groundtruth, the second row are the predictions from the network. The third row are the error between groundtruth and segmentation prediction, where blue region denotes over segmented and red region indicates under segmented.

**Table 1:** Dice scores for LV-Quan segmentation performance.

Model	LV cavity	LV myocardium	Background
UNet	0.950	0.873	0.988
MC-Seg	0.951	0.870	0.986
Densenet	0.957	0.886	0.989
IndicesNet	0.978	0.878	0.988
DRUNet	0.959	0.886	0.989
Parallel	0.966	0.917	0.990
SAUNet	0.969	**0.921**	0.990
Ours	** 0.971**	**0.921**	0.990

Significant values are in bold.

**Table 2:** Hausdorff distance for LV-Quan segmentation performance.

Model	LV cavity	LV myocardium	Background
UNet	5.22	6.69	6.10
MC-Seg	5.22	6.73	6.18
Densenet	3.56	5.43	4.88
IndicesNet	4.12	5.96	5.23
DRUNet	3.55	5.43	4.88
Parallel	**2.85**	3.40	3.29
SAUNet	2.86	**3.31**	3.29
Ours	2.86	3.35	**3.26**

Significant values are in bold.

**Figure 6:**
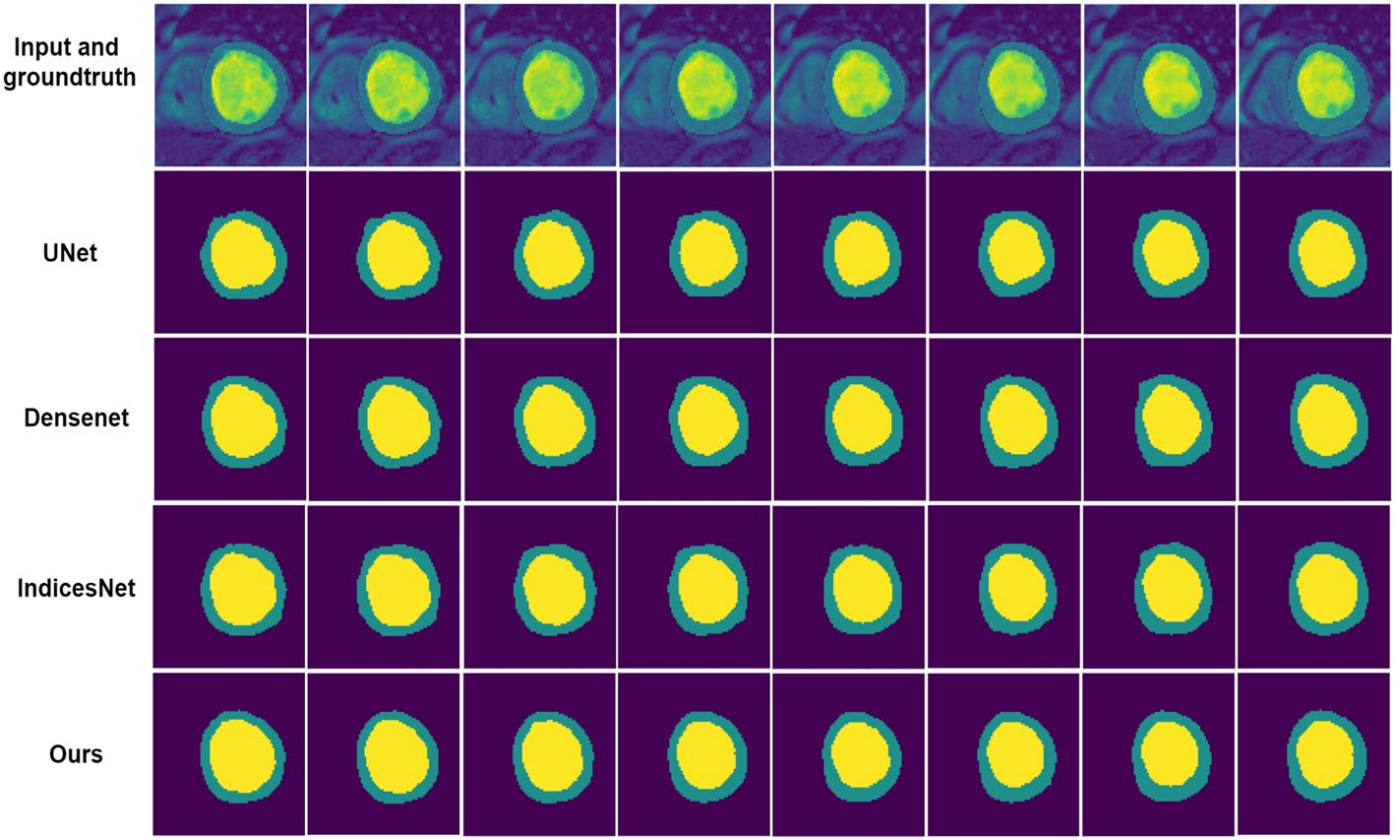
Example of comparative segmentation results on LV-Quan validation data. The first row are the raw input images and its corresponding ground truth. From the second to the fifth row indicate the predictions by comparative ablation models.

**Figure 7:**
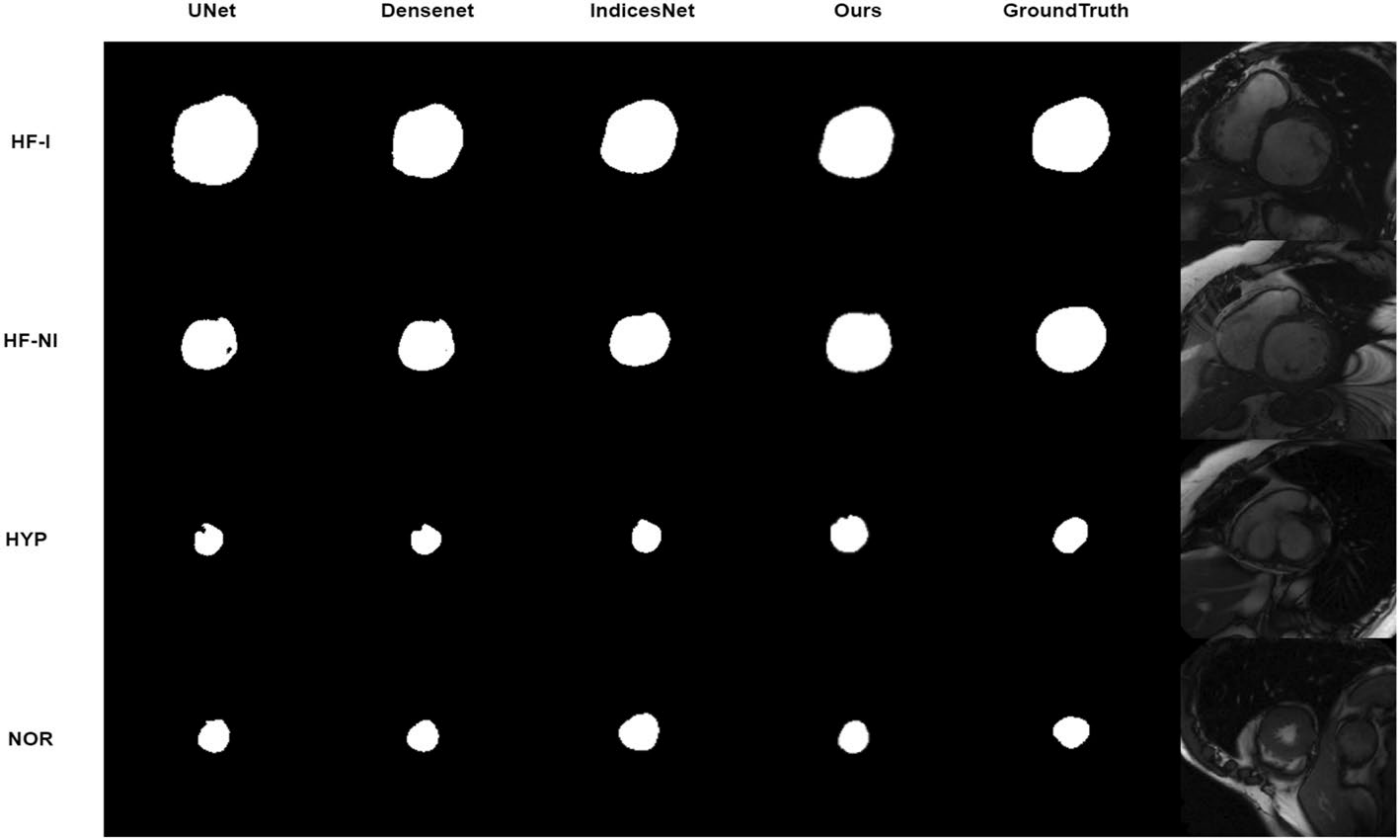
Example of comparative segmentation results on LV-09 testing dataset. The rows from top to bottom indicate the image slices from four pathological group: Heart Failure with Ischemia (HF-I), Heart Failure without Ischemia (HF-NI), Hypertrophic endocardium (HYP), Normal (NOR). The columns from left to right indicate predictions of comparative methods, GroundTruth and raw image data respectively.

We also reproduce classic network to conduct study on segmentation results. Figures 6 and 7 show the analysis on the LV-Quan validation dataset and MICCAI 2009 Sunnybrook Cardiac left ventricle segmentation (LV-09) dataset of our method compared with other classic semantic segmentation networks, such as UNet^49^, Densenet^50^and IndicesNet^38^. Each model is trained for 500 epoches with a batch size of 20, supervised by same loss function and shares the same initial weight of CNN. The hyper-parameter configuration is shared by the selected models. The LV-09 dataset contains 45 cardiac cine-MR short axis (SAX) images from four different pathological groups. Each patient had manually drawn LV endocardium contours for ED and ES slices^54^. In this study, we segment the endocardium as binary boundary, to distinguish anatomical structure between LV and background. The comparative models share the same training strategy. In Fig. 6, for the rows from second to fifth, the Dice coefficient of LV cavity segmentation is 0.940, 0.954, 0.960 and 0,971. The Dice coefficient of LV myocardium segmentation is 0.869, 0.871, 0.880 and 0.921. Figure 7 illustrates the segmentation results on LV-09 dataset. The rows from top to bottom indicate the image slices from four pathological group: Heart Failure with Ischemia (HF-I), Heart Failure without Ischemia (HF-NI), Hypertrophic endocardium (HYP), Normal (NOR). The columns from left to right indicate prediction of comparative methods, ground truth and raw image data. For the columns from left to right, the Dice coeffificient of segmentation is 0.923, 0.932, 0.938 and 0.941. The predictions on HYP patients can best reflect the differences between different models. All above comparative models achieve competitive segmentation performance on HF-NI, HF-I and NOR patients. From the view of Figs. 6 and 7, UNet gets the worst segmentation prediction. The segmentation predictions of our model are the most closely resemble the ground truth. From the results shown in Table 3, UNet is the most time-saving method, and our unified framework is also a time-efficient approach with competitive segmentation performance. Moreover, the number of parameters and computation cost GPU memory usage are the highest for IndiceNet, and the lowest for UNet. When compared to IndicesNet, Densenet uses relatively low parameters and GPU memory to achieve better time-efficient. Since our unified framework contains more convolutions and channels, our model have more parameters than UNet and Densenet, but our GPU memory usage is still relatively small when compared with the IndicesNet.

**Table 3:** Comparison with different methods, the total time of training and testing on the LV-09 and LV-Quan dataset.

Models	LV-Quan		LV-09		Model-complexity	
	Training time (h)	Testing time (s)	Training time (h)	Testing time (s)	Params (M)	GPU memory (G)
UNet^49^	0.4	19	1.5	24	1.43	0.59
Densenet^50^	0.5	23	1.5	20	4.12	1
IndicesNet^38^	0.4	20	1.6	23	39.09	1.2
Ours	0.4	19	1.5	19	33.10	1.11

We also compared the model-complexity of different models. h: hour, s: second.

#### Performance of LV quantification path

Quantification of LV indices is the ultimate purpose of our work. We compare our method with the existing advanced methods (Max Flow^55^, MultiFeatures^29^, SDL^56^, Indices-Net^38^, FullLVNet^57^, DMTRL^39^, Indices-JSQ^6^ and DRUNet^7^) to evaluate the performance. We also add a comparative model to explore the performance difference between segmentation-based model and our task-unified model. The comparative model is the direct morphological calculation method (Calculation), which directly calculate these indices from the segmentation results, not using some simplified regression network. The Calculation model calculate the two Area-myocardium and Area-cavity indices by counting the number of pixels enclosed by endo and epicardium respectively, calculate the three Dim indices by casting a line from the centroid of LV cavity in IS-AL, I-A and IL-AS directions and measuring the distance between the intersections of the casted lines and the LV endocardium contour, and calculate the IS, I, IL, AL, A and AS by casting a line in six directions and measuring the distance between the intersections of the casted line and myocardium. The performance is illustrated in Table 4. Max Flow is is a multi-step model based on indirect-segmentation method, which LV quantification indices are calculated by the LV myocardial contour segmented first. The Max Flow method has high MAE of LV regional wall thicknesses metrics, but the PCC metrics are better than that of some direct methods. The reason is that, this method calculate LV indices by extracted contour, which results in a better mapping to label. The calculation method is also a indirect-segmentation method, which gets poor MAE and PCC performance compared with direct regression methods. Multi-features and SDL are two-step direct regression methods, they learn the cardiac image features first, and then use the representative features to quantify LV indices. In Table 4, we can conclude that the two-step direct regression methods get poor performance not only in high MAE but also in correlation with the ground-truth. The poor representation ability of two-step methods result in high MAE and low PCC values. Indice-Net is an end-to-end manner foundation method to predict LV indices. Compared to Max Flow, Indices-Net gets a better area MAE metric but a poor regional wall thickness MAE metric. FullLVNet and DMTRL utilized RNN module to capture dynamic information which further improve the quantification results. The Indices-JSQ leveraged segmentation predictions to calculate the LV indices. DRUNet introduced a multi-task learning approach to regress the cardiac LV indices. It can be seen in experimental results, direct methods outperform most indirect methods. Our method yields average MAE values of 132 mm^2^, 1.78 mm, 1.16 mm for area, cavity dimension, and regional wall thickness. The average PCC values of area, cavity dimension, and regional wall thickness are 0.962, 0.978, 0.872 , respectively. Our task-unified network is an end-to-end manner, which incorporate the advantages of indirect and direct methods to improve segmentation predictions supervised by indices of label and generate more accurate quantification LV indices.

**Table 4:** Comparison with state-of-the-art methods of the quantification performance.

Indices	Metrics	MaxFlow	Multi-features	SDL	FullVNet	DMTRL	Indice-Net	Indice-JSQ	DRUNet	Calculation	Ours
A-cav	MAE	156 ± 193	231 ± 193	198 ± 169	181 ± 155	172 ± 148	185 ± 162	157 ± 145	106 ± 87	163 ± 130	105 ± 90
	PCC	0.958	0.924	0.942	0.940	0.943	0.953		0.985	0.952	0.985
A-myo	MAE	339 ± 272	291 ± 246	286 ± 242	199 ± 174	189 ± 159	223 ± 193	157 ± 161	165 ± 132	197 ± 175	158 ± 130
	PCC	0.851	0.729	0.742	0.935	0.947	0.853		0.935	0.913	0.938
Average	MAE	247 ± 201	261 ± 165	242 ± 158	190 ± 128	180 ± 118	204 ± 133	157 ± 120	135 ± 29	180 ± 159	132 ± 110
	PCC	0.904	0.827	0.842	0.937	0.945	0.903		0.960	0.933	0.962
Dim1	MAE	2.81 ± 2.76	3.53 ± 2.77	2.99 ± 2.43	2.62 ± 2.09	2.47 ± 1.95		2.43 ± 1.91	1.76 ± 1.43	2.61 ± 2.24	1.80 ± 1.52
	PCC	0.937	0.885	0.914	0.952	0.957			0.975	0.937	0.973
Dim2	MAE	2.64 ± 2.12	3.49 ± 2.87	2.55 ± 2.30	2.62 ± 2.09	2.59 ± 2.07		2.32 ± 1.77	1.80 ± 1.49	2.67 ± 2.58	1.79 ± 1.49
	PCC	0.946	0.897	0.938	0.881	0.894			0.977	0.938	0.977
Dim3	MAE	2.49 ± 2.88	3.91 ± 3.23	3.10 ± 2.54	2.77 ± 2.22	2.48 ± 2.34		2.54 ± 1.97	1.72 ± 1.41	2.57 ± 2.31	1.75 ± 1.38
	PCC	0.945	0.865	0.903	0.935	0.0.943			0.978	0.933	0.973
Average	MAE	2.65 ± 2.33	3.64 ± 2.61	2.88 ± 2.03	2.68 ± 1.64	2.51 ± 1.58		2.43 ± 1.62	1.76 ± 1.44	2.61 ± 2.38	1.78 ± 1.46
	PCC	0.943	0.882	0.910	0.917	0.925			0.977	0.936	0.978
IS	MAE	1.53 ± 1.73	1.70 ± 1.47	1.98 ± 1.58	1.32 ± 1.09	1.26 ± 1.04	1.39 ± 1.132	1.16 ± 1.03	1.15 ± 0.93	1.55 ± 1.49	1.12 ± 0.88
	PCC	0.796	0.729	0.611	0.840	0.856	0.824		0.0.908	0.872	0.913
I	MAE	3.23 ± 2.83	1.71 ± 1.34	1.67 ± 1.40	1.38 ± 1.10	1.40 ± 1.10	1.51 ± 1.21	1.33 ± 1.07	1.24 ± 1.01	1.89 ± 1.93	1.28 ± 1.02
	PCC	0.720	0.603	0.462	0.751	0.747	0.701		0.856	0.700	0.851
IL	MAE	4.15 ± 3.17	1.97 ± 1.54	1.88 ± 1.63	1.57 ± 1.35	1.59 ± 1.29	1.65 ± 1.36	1.42 ± 1.20	1.42 ± 1.13	1.92 ± 1.53	1.35 ± 1.13
	PCC	0.743	0.483	0.435	0.691	0.693	0.671		0.836	0.703	0.811
AL	MAE	5.08 ± 3.95	1.82 ± 1.41	1.87 ± 1.55	1.60 ± 1.36	1.57 ± 1.34	1.53 ± 1.25	1.37 ± 1.18	1.37 ± 1.08	1.96 ± 1.67	1.34 ± 1.11
	PCC	0.706	0.533	0.547	0.651	0.659	0.698		0.829	0.660	0.820
A	MAE	3.47 ± 3.25	1.55 ± 1.33	1.65 ± 1.45	1.34 ± 1.11	1.32 ± 1.10	1.30 ± 1.12	1.21 ± 1.07	1.13 ± 0.97	1.62 ± 1.55	1.08 ± 0.91
	PCC	0.724	0.685	0.661	0.768	0.777	0.781		0.875	0.777	0.880
AS	MAE	1.76 ± 1.80	1.68 ± 1.43	2.04 ± 1.59	1.26 ± 1.10	1.25 ± 1.01	1.28 ± 1.00	1.24 ± 1.08	1.05 ± 0.84	1.58 ± 1.33	1.11 ± 0.94
	PCC	0.785	0.777	0.726	0.864	0.877	0.871		0.928	0.854	0924
Average	MAE	3.21 ± 1.98	1.73 ± 0.97	1.85 ± 1.03	1.41 ± 0.72	1.39 ± 0.68	1.44 ± 0.71	1.29 ± 0.70	1.23 ± 1.01	1.75 ± 1.58	1.16 ± .0.99
	PCC	0.785	0.777	0.726	0.864	0.877	0.871		0.928	0.761	0.924
Phase	ER				10.4	8.2			10.8		9

MAE and PCC are shown in table.

**Figure 8:**
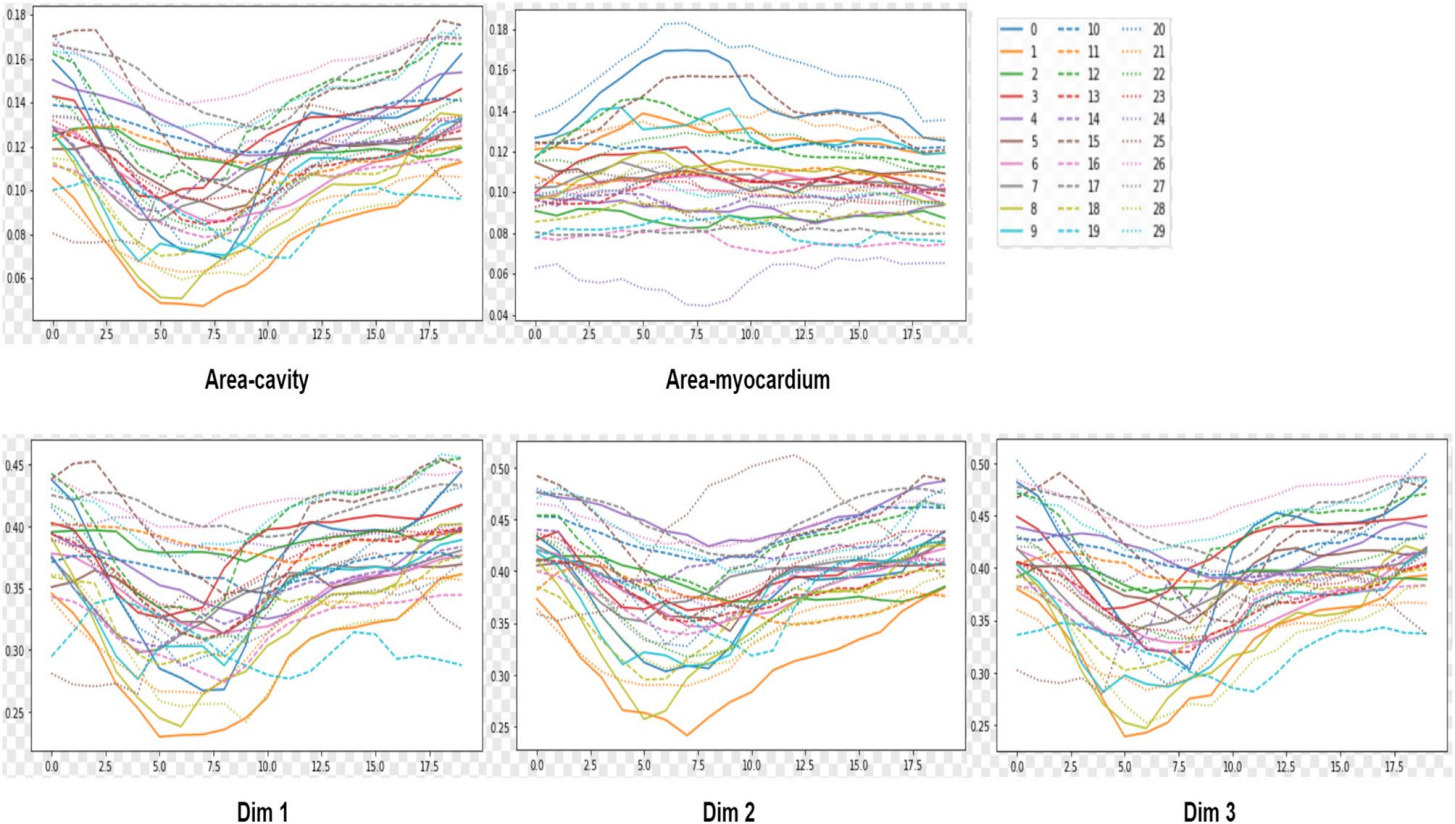
The LV quantification indices results predicted by our method on the testing data.

**Figure 9:**
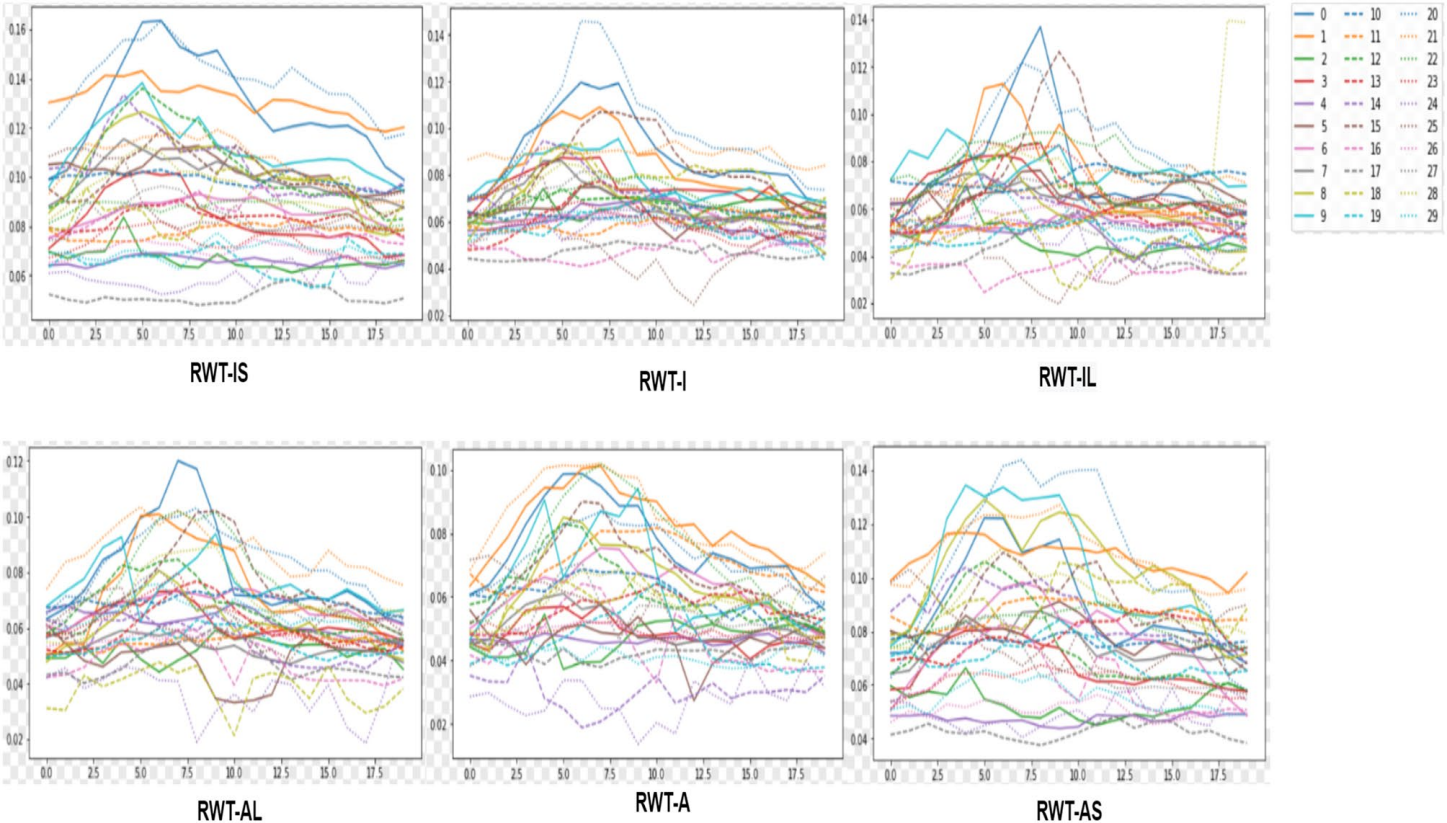
The LV quantification indices results predicted by our method on the testing data.

**Figure 10:**
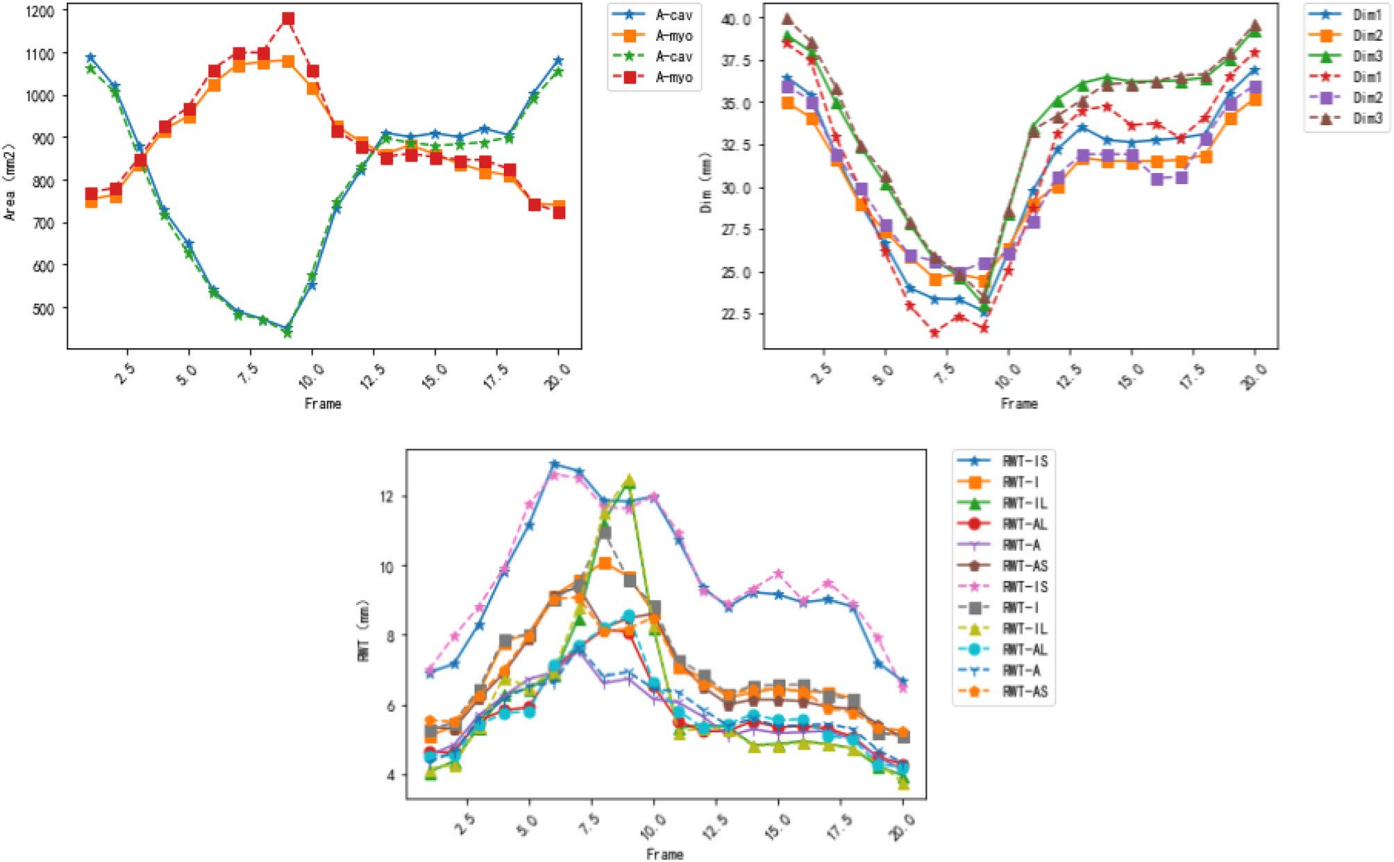
Examples of LV metrics predicted by our unified network for a random patient during a cardiac cycle (20 frames). In the following line charts, the predicted results are shown with the dotted line and the corresponding ground truth values are displayed with the solid line in the same marker.

We evaluate our unified framework on the testing data. Figures 8 and 9 show the normalized results of the quantification indices. The values of RWT, dimension and areas are normalized. Figure 10 illustrates the clinical metrics results of a randomly patient data subject compared with quantification metrics predicted by our task-unified model. In every image, the dotted line in the figure is the quantification prediction result of our task-unified model, and the solid line shows the metrics of groundtruth. Seen from the prediction results of clinical metrics, they are very close to the annotated label. The three figures of experimental result illustrate that our unified network achieves competitive performance in LV indices quantification.

**Figure 11:**
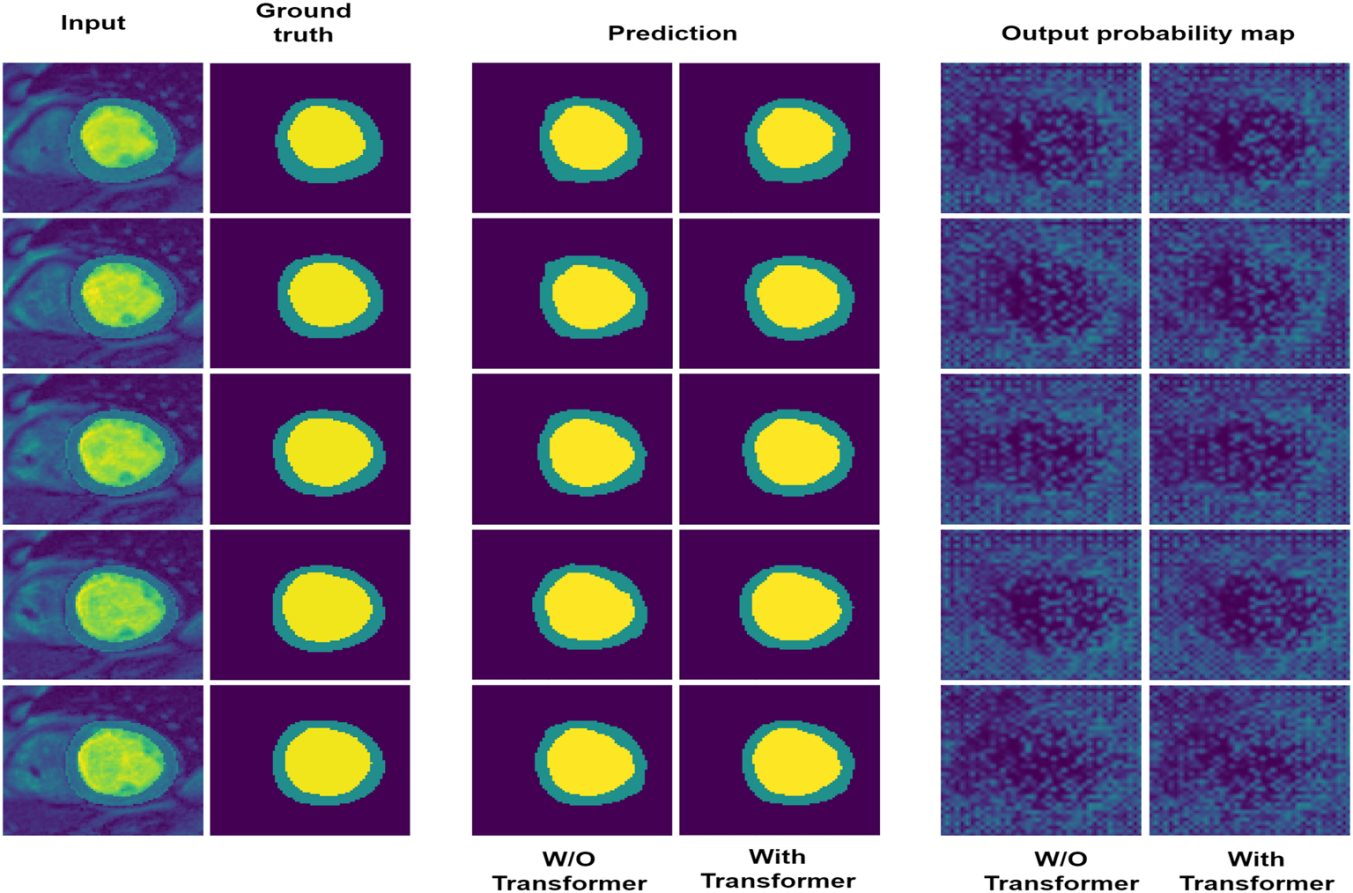
Visualization of prediction and output probability map. The rows marked W/O transformer indicates the model without using transformer module, while with transformer indicates model using transformer module.

To better understand the ability of feature extraction of transformer, we conduct ablation study by using two models, and visualize the prediction and output probability map in Fig. 11. One ablation model is our proposed unified network, another ablation model is a simplified version of our model which removed the transformer block. It can be seen that the prediction and output probability map of simplified model are more blurred, the segmentation path with transformer module predicts more concise results. Thus to prove the extraction ability of transformer.

## Conclusions

In this study, we introduce a accurate and efficient deep learning segmentation and regression unified network to segment and quantify the LV simultaneously. The segmentation module leverage an U-Net like 3D Transformer model to predict the contour of three anatomy structures, while the regression module learned spatial–temporal representations from the original images and the reconstruct feature maps from segmentation path to estimate the finally desired quantification metrics. The three anatomy structures contains LV cavity, LV myocardium and background. The quantification metrics including the LV myocardial RTWs, dimensions, cavity and myocardium areas, and the cardiac diastolic or systolic phase. We used a joint-task loss function to supervise the two module networks training approach. Although the LV anatomical shape and appearance are highly variable across different subjects, our model achieves competitive performance in both segmentation and quantification approach. The unified network was evaluated on MICCAI 2017 LV-Quan dataset, and the experimental results prove the accuracy and efficiency of our model . In the future, we will verify our framework on more datasets to test the contribution in clinical approach.

## Data Availability

The LV-Quan data is available at https://lvquan18.github.io/, the Sunnybrook data is available at https://www.cardiacatlas.org/sunnybrook-cardiac-data/.
